# Supplementary figures and images for: Classifying Breast Cancer Subtypes Using Deep Neural Networks Based on Multi-Omics Data
Source: Genes (Basel). 2020 Aug 4;11(8):888. doi: 10.3390/genes11080888 (PMC7464481; doi:10.3390/genes11080888)

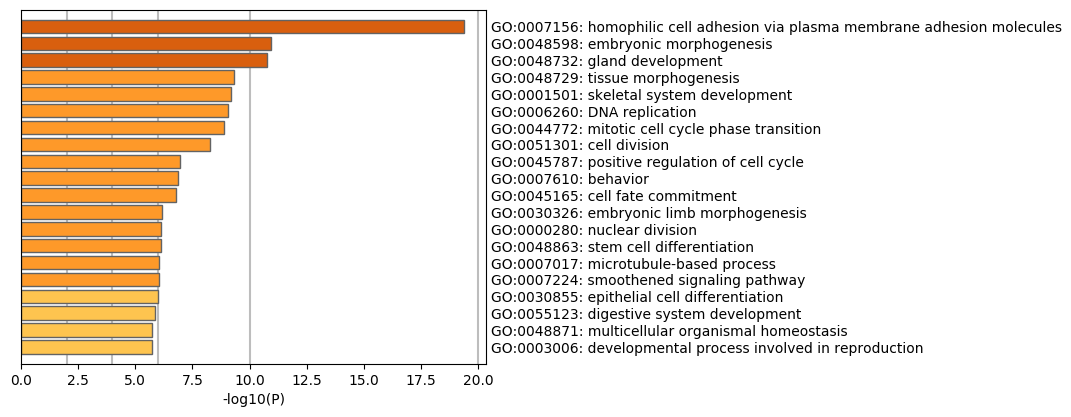

Supplement: Supplementary file 1 [file genes-11-00888-s001.zip › Supplementary Materials/Figure S1:Bar Graph of GO_BP.png]

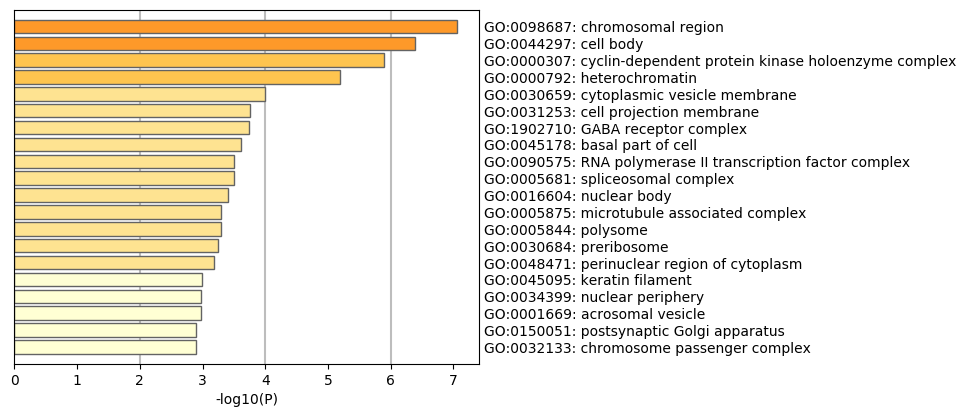

Supplement: Supplementary file 1 [file genes-11-00888-s001.zip › Supplementary Materials/Figure S2:Bar Graph of GO_CC.png]

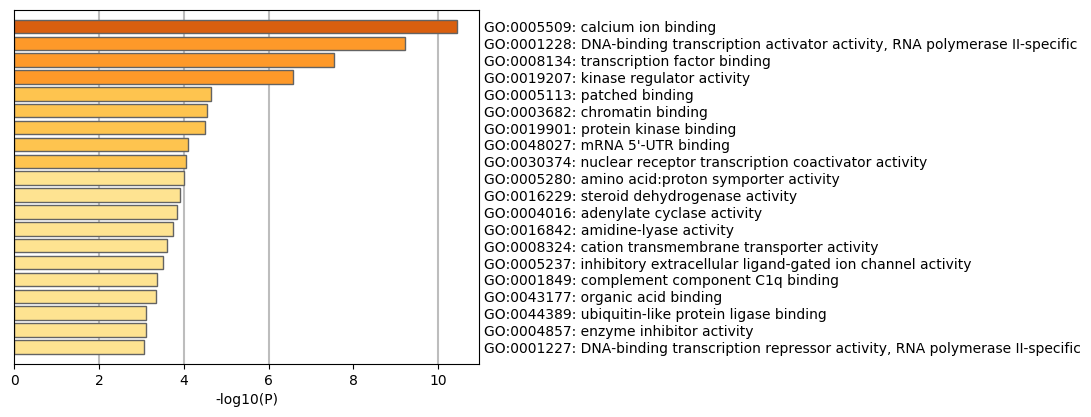

Supplement: Supplementary file 1 [file genes-11-00888-s001.zip › Supplementary Materials/Figure S3:Bar Graph of GO_MF.png]
